# Nimotuzumab in the Treatment of Inoperable Esophageal Tumors of Epithelial Origin

**DOI:** 10.1155/2022/4128946

**Published:** 2022-09-01

**Authors:** Sandra González Fernández, Yohan Amador García, Lucien Gregoria Boris Porras, Liuba Mojena Martínez, Luis Laureano Soler Porro, Gustavo Pish Martí, Liem Fonseca Chon, Yelec Estrada Guerra, Jorge Manuel Álvarez Blanco, Acralys Garabito Perdomo, Félix Bárbaro Santel Odio, Luis Alfonso Varona Vázquez, Yoel Mario Ricardo Serrano, Lisette Chao González, Yisel Avila Albuerne, Lizet Sánchez Valdés, Carmen Elena Viada González, Mayra Ramos Suzarte, Yaimarelis Saumell Nápoles

**Affiliations:** ^1^Conrado Benítez Hospital, Libertadores & P. Martí, CP: 90100, Santiago de Cuba, Cuba; ^2^José Ramón López Tabranes Hospital, Carretera Central Km. 101, CP. 40 100, Matanzas, Cuba; ^3^III Congreso Hospital, 2da y 26 de Julio, Pinar Del Río, Cuba; ^4^Vladimir Ilich Lenin Hospital, 4 Lenin Avenue, Holguín, Cuba; ^5^Manuel Ascunce Domenech Hospital, Carretera Central Oeste Km 4 1/2, Camagüey, Cuba; ^6^Celia Sánchez Manduley Hospital, Avenida Camilo Cienfuegos Km 1, Vía Campechuela, CP: 87510, Manzanillo, Granma, Cuba; ^7^Ernesto Guevara de La Serna Hospital, Ave. 2 Diciembre, No. 1, CP:75100, Las Tunas, Cuba; ^8^Antonio Luaces Iraola Hospital, Calle Máximo Gomez, No. 257, e/ 4ta y Onelio Hernández, CP: 65200, Ciego de Ávila, Cuba; ^9^Camilo Cienfuegos Hospital, Bartolomé Masó, No. 128, CP. 60 100, Havana, Sancti Spíritus, Cuba; ^10^Celestino Hernández Robau Hospital, Avenida Liberación, No. 99, Santa Catalina, CP. 50100, Santa Clara, Villa Clara, Cuba; ^11^Agosthino Neto Hospital, Carretera El Salvaor Km 1, CP: 95100, Guantánamo, Cuba; ^12^Gustavo Aldereguía Lima Hospital, Ave 5 de Septiembre y Esq. Calle 51-A, CP: 55100, Cienfuegos, Cuba; ^13^Carlos Manuel de Céspedes Hospital, Carretera Km1 Vía Santiago de Cuba, CP: 85100, Bayamo, Granma, Cuba; ^14^Medical and Surgical Research Center (CIMEQ), Calle 216 y 11B, Reparto Siboney, Havana, Cuba; ^15^Cuba's National Clinical Trials Coordinating Center, 60 & 5th Avenue, Playa, Havana, Cuba; ^16^Center of Molecular Immunology, 216 & 15 Street, Atabey, Havana, Cuba

## Abstract

**Background:**

Nimotuzumab is a humanized monoclonal antibody that targets the epidermal growth factor receptor. It was approved in Cuba for the indication of inoperable malignant tumors of the esophagus of epithelial origin. The purpose of this study was to evaluate the safety, overall and progression-free survival, clinical response, and quality of life, in adult patients with inoperable esophageal tumors of epithelial origin treated with nimotuzumab in a practical context. *Material and Methods*. The number of patients who developed adverse events was determined, and the frequency, seriousness, causality, and severity of these adverse events were determined. It also determined the median of survival and progression-free survival and rates at 12 and 24 months and the quality of life.

**Results:**

A total of 111 patients were included. The proportion of serious and related AE with the use of nimotuzumab was 1.3%. Most of the related AEs were mild and moderate, and the most frequent AEs were diarrhea, chills, and tremors. New diagnosed patients who received nimotuzumab concurrent with chemotherapy and radiotherapy reached a median OS of 12.2 months (95% CI, 6.9–17.5) and 12- and 24-month survival rates of 51.0% and 17.0%, respectively. Median PFS was 7.8 months (95% CI, 6.2–9.5), and 12- and 24-month PFS rates were 39.3% and 11.2%, respectively. A favorable evolution of the general state of health (*p*=0.03) was obtained from the beginning of treatment until month 12, with a significant reduction in the appearance of nausea (*p*=0.009), insomnia (*p*=0.04), constipation (*p*=0.04), eating difficulties (*p*=0.0006), and choking when swallowing (*p*=0.0001), but increased in dysphagia (*p*=0.02).

**Conclusions:**

The administration of nimotuzumab was safe in the real-world setting. New diagnosed patients that received nimotuzumab concurrent with chemotherapy and radiotherapy reached a higher overall and progression-free survival and better quality of life than the rest of the patients. Trial registration is RPCEC00000215 (Cuban Registry of Clinical Trials; https://registroclinico.sld.cu/en/home). It is registered prospectively on June 30, 2016.

## 1. Introduction

In 2018, esophageal cancer (among 36 most frequent types of cancer) ranked 7^th^ worldwide in terms of incidence (572,034 new cases for 3.2%) and 6^th^ place in mortality (508,585 deaths for 5.3%). Approximately 70% of cases were male ones, with incidence and mortality rates multiplying between 2 and 3 times compared with female sex [1]. In Cuba in 2016, 704 new male cases were reported, which represented a gross rate of 12.6 × 100 000 inhabitants and 7.9 of the age-adjusted rate for the world population, while in the female sex it does not appear among the top ten locations with the highest incidence. According to this report, in 2019 there were 787 deaths from esophageal tumors, reaching a rate of 7.0 per 100,000 habitants [2].

Treatment strategies for esophageal cancer include chemotherapy (CT), radiotherapy (RT), and surgery, being more aggressive over the years. After exploratory surgery, surgical resection is finally possible in only 15–20% of patients. In less than 60% of patients with locoregional disease, curative resection is achieved. Approximately 70–80% of those patients undergoing surgery have regional lymph node metastases, which is a strong predictor of poor survival. In patients treated with surgery alone, 5-year survival of about 34–50% is reported. Although surgery is also used for palliative purposes in the presence of dysphagia or fistulas, its use is not recommended in patients with inoperable or advanced cancer who presents comorbidities such as severe heart or lung disease. RT, as well as monotherapy, is used only as palliative treatment, or in those patients who are not fit for CT. For patients who are not candidates for surgery, doses of 50.0 to 50.4 Gy (divided into fractions of 1.8 to 1.8 Gy per day) are recommended, so lower doses are not adequate [3].

The most frequently used CT agents are 5-fluorouracil (5-FU), cisplatin, and taxanes, which have shown encouraging response rates and manageable toxicity. The indication of cisplatin concurrent with 5-FU is one of the most recognized for these patients. The combination of neoadjuvant CT and neoadjuvant RT is more efficient than RT as a single treatment in patients with non-resectable locally advanced or metastatic esophageal cancer and may increase control of local or disseminated disease. This combination has two advantages: CT can overcome metastasis that falls outside the radiation field, and the local efficacy of RT can be increased by a radiosensitization effect in the case of concomitant CT, or by a reduction in tumor size in the case of sequential CT [3].

Partly owing to the late diagnosis, the prognosis of esophageal cancer has been dismal regardless of the aggressive treatments. Therefore, treatment should be based on tumor stage, patient's physical performance, tolerance of the treatment, and histology. Notwithstanding, in randomized clinical trials few and still no consistent benefit has been observed for any specific approach. Esophageal cancer and its everyday recurrences are more resistant to available treatments, with no beneficial results when assessing overall survival. Immunotherapy has been explored for some years as another modality under evaluation for its treatment [4].

The epidermal growth factor receptor (EGFR) is a transmembrane tyrosine kinase receptor (RTK) and a representative target for cancer therapy to which small molecule tyrosine kinase inhibitors (TKIs) and monoclonal antibodies (mAbs) have been developed, approved, and applied [5]. Overexpression of EGFR in esophageal squamous cell carcinoma (ESCC) patients varies between 33.3 and 72.1%. This overexpression and the amplification have been significantly correlated with advanced tumor stage, tumor invasion, the occurrence of metastasis, involvement of lymph node, and poor survival outcome [6, 7].

Nimotuzumab is a humanized mAb that targets and binds to the EGFR with intermediate affinity (lower than other monoclonal antibodies) since its dissociation constant is intermediate. It requires bivalent binding for a stable union, and it will selectively bind from moderate to high EGFR-expressing cells. Furthermore, when EGFR density is low as in normal tissue, the interaction is transient [8]. Nimotuzumab has antiproliferative, proapoptotic, and antiangiogenic effects. It can also induce natural killer (NK) cell activation, antibody-dependentcell-mediated cytotoxicity (ADCC), dendritic cell (DC) activation, and upregulation induction of programmed death/ligand 1 (PD-L1) molecule on DCs [9, 10]. Evidence of low toxicity and efficacy of nimotuzumab have been previously documented for esophageal cancer [11–16] and other indications [17–22].

Since 2010, the monoclonal concurrent with CT and RT was approved for the indication of inoperable malignant tumors of the esophagus of epithelial origin, by the Regulatory Authority for Medicines and Medical Devices of Cuba (CECMED, by its Spanish acronym).

## 2. Materials and Methods

### 2.1. Study Design

A phase IV, uncontrolled, open, and multicenter clinical trial was designed with the objective of evaluating adverse events, OS and PFS, clinical response, and quality of life (QoL), in patients with inoperable tumors of the esophagus of epithelial origin. These were treated with nimotuzumab + CT + RT, nimotuzumab + CT, nimotuzumab + RT, or nimotuzumab as monotherapy. The first patient was enrolled on 15/07/2016 and the last was on 10/07/2018. All patients were treated with 200 mg of nimotuzumab intravenously (IV) once a week for 6 weeks, and subsequently regardless of the clinical response achieved, every 14 days until interruption due to any of the causes provided for in the protocol. If the patient was medically fit for RT, he concurrently received 50.4 Gy in no more than 30 sessions, indicating 1.8 Gy daily, 1–5 days a week, Monday through Friday, from the 2^nd^ to the 7^th^ weeks.

If the patient was medically fit for CT, he received it concomitantly considering 2 strata: the first formed by new diagnosis patients, who received the CT of 1st line from the 2nd week, every 28 days (4 weeks) and for 4 cycles. CT comprised cisplatin (CDDP), 75–100 mg/m^2^, day 1, and 5-fluorouracil (5-FU), 750–1000 mg/m^2^ in continuous infusion, days 1–4 of the week. At 18 weeks, the clinical antitumor response was evaluated. In patients with partial response, stable disease, or progression, the 2^nd^ line of CT was also indicated as it is established in the treatment standards and considering the availability in each institution. In the second stratum, patients were included with disease recurrence at the time of inclusion. They received treatment with nimotuzumab, concomitant with 2^nd^ line CT as is established in the treatment standards and considering the availability of cytostatics in each institution. If a subsequent imaging evaluation detected disease progression, another 2^nd^ line CT was indicated.

Diagnostic criteria are as follows: patients with histological diagnosis of esophageal tumor (locally advanced, recurrent, or metastatic) of epithelial origin, located in the cervical or intrathoracic esophagus (middle or upper portion).

Inclusion criteria are as follows: patients of ≥18 years old who meet the diagnostic criteria and express written willingness to participate in the study with the signature of informed consent, with life expectancy equal to or greater than 6 months and clinical status according to ECOG criteria ≤2.

Exclusion criteria are as follows: pregnancy, puerperium, or lactation; patients with a second concomitant tumor (with the exception of basal or squamous carcinoma of the skin and treated carcinoma in situ of the cervix), or cerebral metastasis diagnosis by computerized tomography, uncontrolled, or in progression; the presence of chronic or uncontrolled comorbidities, allergic states or septic, acute or severe processes, and history of hypersensitivity to any component of the nimotuzumab formulation; or patients who received prior treatment with nimotuzumab or other biological therapy 6 months before inclusion, or is receiving another research product.

### 2.2. Ethical Considerations

The study was conducted in agreement with the general principles adopted by the international community regarding biomedical research in human subjects, with current state regulations according to the requirements of the Cuban national regulatory agency, as well as in the Guide to Good Clinical Practices of the International Harmonization Conference (ICH E6). Furthermore, it was approved by the Cuban Minister of Public Health, the Institutional Ethics Committees of each hospital, and CECMED. The informed written consent was obtained from the patients before their inclusion in the investigation.

### 2.3. Evaluation during the Study

The study considered a treatment period of two years, during which patients were evaluated from the clinical point of view at the beginning of treatment (baseline time), before each cycle of chemotherapy at 18 weeks, and, thereafter, every three months. OS and PFS were estimated for all patients. OS was defined as the time from randomization until death from any cause, and PFS, from randomization until progression.

The scores of two quality-of-life questionnaires (QLQ) of the European Organization for Research and Treatment of Cancer (EORTC) were estimated at baseline and every 3 months: QLQ-C30 (version 3) and the specific for esophageal cancer QLQ-OES18 (version 3).

### 2.4. Statistical Analysis

The optimal number of subjects to include should be 104 patients for a level of significance *α* = 0.05 and potency *β* = 0.80. However, it was decided to include all the patients recruited during two years (the inclusion period). All analyses were done in the intention-to-treat population. To estimate the number of subjects required were used the NCSS Trial, PASS 2005, and GESS 2006 software, in the power analysis of one proportion.

The frequency (percentage, 95% confidence interval, CI) of patients who developed AE was determined, and the AEs were described in terms of seriousness (serious/nonserious), intensity (by using the Common Toxicity Criteria, version 4.0), and causality (related/not related). For the survival variables was used the Kaplan–Meier, and were determined the median and survival rates at 12 and 24 months. For clinical response were used frequency tables. The generalized linear model and the generalized estimation equations were used for the QoL longitudinal analysis.

## 3. Results

### 3.1. Patient Population

**Figure 1:**
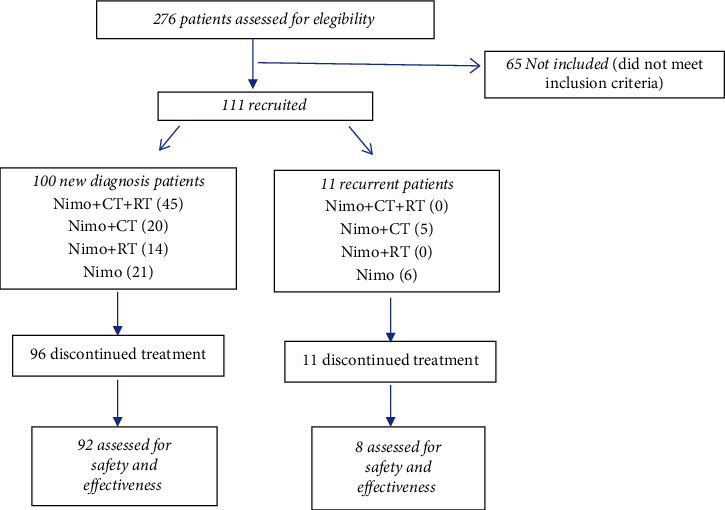
Distribution of study patients.

Between August 22, 2016, and July 30, 2018, 276 patients were evaluated. Of these, 111 were included in the study (Figure 1). Only 67 patients (60.4%) completed the induction treatment (six doses) and went on to maintenance, 61 of these in the new diagnosis stratum. 11 patients (9.9%) did not receive any monoclonal dose.

71% of new diagnosis patients received six or more doses of the monoclonal. 39 (54.9%) of them received nimotuzumab + CT + RT. In the recurrence stratum, eight patients received six or more doses of the monoclonal. The main causes of early dropout were death (28.04%), rapid worsening of the performance status (27.10%), and consent withdrawal (21.50%).

In general, a male predominance was observed. The mean age was 60 years for patients treated with concurrent Nimo + CT + RT and near this value for the rest (Table 1). In the new diagnosis stratum, patients with tumor location in the middle intrathoracic region and the clinical stage IIIb predominated in all treatment groups. In the stratum of recurrent patients, the behavior was similar.

### 3.2. Safety Results

In the evaluated population, at least some AEs were reported in 61 patients (61%). A total of 229 adverse events of 81 different types were recorded. The AEs related to treatment were recorded in 6.1% of patients. AEs with grade 3 (6.1%) and grade 4 (3.9%) of intensity were reported in 17% of the patients who had AE. Serious AEs related to treatment were recorded in 27% of patients; of these, only 1.3% were related to the monoclonal (Table 2).

Anorexia, dysphagia, weight loss, anemia, and vomiting appeared more frequently than 5%. The most frequent monoclonal-related adverse events were diarrhea, chills, and tremors with 0.9% each one. The majority of the patients presented moderate and mild AE.

### 3.3. Effectiveness Results

**Figure 2:**
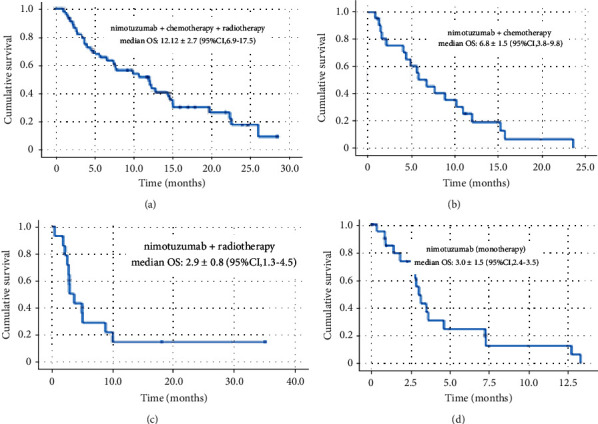
Kaplan–Meier curves for overall survival in new diagnosis patients.

Figure 2 shows the Kaplan–Meier curves for the overall survival in the new diagnosis stratum. Patients who received nimotuzumab + CT + RT reached a median OS of 12.2 months (95% CI, 6.9–17.5), and the 12-month OS rates were 51%. Only this group reached a 24-month survival rate, with a value of 17.2%. Patients treated with Nimo + CT reached a median OS of 6.8 months (95% CI, 3.8–9.8), and the 12-month OS rates were 18.8%. Patients treated with nimotuzumab + RT and nimotuzumab as monotherapy did not reach a 12-month OS rate.

**Figure 3:**
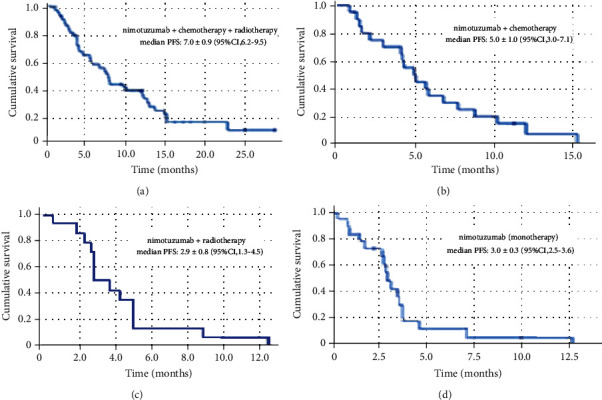
Kaplan–Meier curves for the progression-free survival in the new diagnosis stratum.

Figure 3 shows the Kaplan–Meier curves for the progression-free survival in the new diagnosis stratum. New diagnosis patients receiving nimotuzumab concurrent with chemotherapy and radiotherapy reached a progression-free survival of 7.8 months (95% CI, 6.2–9.5), and a progression-free survival rate at 12 and 24 months was 39.3 and 11.2%, respectively. Patients treated with nimotuzumab and chemotherapy reached a 12-month survival rate of 7.5%, but they did not reach the 24-month survival rate. Patients treated with nimotuzumab + RT and nimotuzumab as monotherapy did not reach a 12-month OS rate.

Of 11 patients included in the stratum of recurrent disease, the subgroup of those treated with nimotuzumab + CT (*n* = 5) reached a median OS of 4.9 months (95% CI: 0.53–9.27) and a 6-month survival rate of 40%, while the subgroup of those treated with nimotuzumab as monotherapy (*n* = 5) reached a median overall survival of 4.9 months (95% CI: 0.31–6.55) and a 6-month survival rate of 16.7%.

As a result of the response analysis (Table 3), 16.0% of complete remission and 8.0% of partial remission were obtained, which resulted in an objective response of 24.0%. 56% of the patients were not evaluated for the response to treatment, because they interrupted the study before 18 weeks, the moment of evaluation provided for in the protocol.

The data from the baseline questionnaire were provided for 87% of new diagnosis patients (Table 4). Of the total patients studied at 6 and 12 months, 36.2% and 42.8% of the patients, respectively, provided questionnaire data.

A favorable evolution of the general state of health of these patients was obtained from the beginning of treatment until month 12, with a significant reduction in the appearance of nausea, insomnia, and constipation.

Table 5 shows the evolution of the quality of life over time for these specific parameters of esophageal cancer, according to the QLQ-EOS18 questionnaire. Difficulty eating and choking when swallowing reached a statistically significant reduction at one year of treatment with respect to the score reached in the baseline measurement. The increase in dysphagia was also significant.

## 4. Discussion

In the international setting, the effectiveness evidence about the treatment of squamous cell esophageal carcinomas is insufficient, and immunotherapy has been explored concurrently with the standard chemotherapy with the purpose of improving at least the disease control [3].

A snag of EGFR-targeted therapies is the severe induced skin rash, lymphocytic infiltrates, folliculitis or perifolliculitis, and other related adverse events that they provoke in renal cells and gastrointestinal mucosa [8, 23, 24]. These side effects are believed to be caused by interaction of the anti-EGFR targeting drugs with the receptor in other tissues than the tumor. Nevertheless, the bivalent binding by intermediate affinity of nimotuzumab provides a pharmacological advantage given by the antitumor activity with preferential uptake in EGFR overexpressing tumors, and low uptake in normal tissues (i.e., the skin and kidney), and consequently, a reduced incidence of toxicities observed with other approved anti-EGFR antibodies, such as the acneiform rash appeared with cetuximab [8, 25].

This study was designed with the purpose of consolidating the nimotuzumab safety profile, and survival effect in the treatment of patients with tumors of the esophagus of epithelial origin (unsuitable for or who refuse surgery) in a real-life setting was evaluated. Nimotuzumab showed the antitumor activity encouraging the clinical results with a very low toxicity profile, without severe skin and renal toxicities. The majority of the related AEs were mild and moderate, regardless of the treatment modality, and evolved towards recovery or improvement. No patient died due to an adverse event associated with the use of the monoclonal. The proportion of serious AE related (very probable, probable, or possible) to the use of nimotuzumab was 1.3% so, was fulfilled the hypothesis raised in the study that the incidence of these events was lower than 10%.

The most frequent and related adverse events (diarrhea, chills, and tremors) that appeared in this study have been reported in previous studies with the monoclonal in different localizations [11–22]. In a phase II carried out by Liang and others, the common acute toxicities during the treatment were esophagitis and blood/bone marrow, dermatological, and gastrointestinal complications. The incidence of grade 3 toxicity was 21.4%; no grade 4 toxicity occurred. Mild, nimotuzumab-related skin rash was observed in four patients but did not require treatment [13]. Castro and colleagues published the safety results of phase II clinical trial that confirmed the safety profile of the combination of nimotuzumab plus chemoradiotherapy, demonstrated in previous clinical trials with this antibody. Toxicity was dominated by nutritional deterioration (12.6% with dysphagia grade 3-4) and side effects associated with chemotherapy [16].

The survival advantage and long-term duration of effect seen after few weeks of treatment with nimotuzumab across different studies and localizations of cancer may be explained by their combined mechanism of action: the inhibition of EGFR signal transduction and tumor proliferation, and the capacity to induce ADCC-mediated tumor cell killing and adaptive immunity through tumor antigen-specific T cells [9].

Different anti-EGFR antibodies or small molecules have been tested in ESCC. Up to date, however, there is little evidence of phase III and post-marketing studies that have shown an important benefit of combining other immunotherapies with chemoradiotherapy for the treatment of locally advanced or metastatic ESCC. Overall survival was lower with targeted agents such as panitumumab and cetuximab than with chemotherapy [26, 27].

In this study, with a maximum follow-up time of 35.2 months and average follow-up time of 7.9 months, a survival benefit was obtained in new diagnosis patients treated with nimotuzumab concurrent with chemotherapy and radiotherapy. Although the treatment groups were not comparable from a statistical point of view, this one reached the following benefits: a median overall survival at least 6 months higher; a 12- and 24-month survival rate higher in 30% and 17%, respectively; and a progression-free survival at least two months higher than for the rest of the patients treated with the monoclonal in another regimen.

The median overall survival of 12.2 months and the 12-month survival rate of 51.0% obtained in this study for patients treated with nimotuzumab plus CT-RT were similar to that obtained in the comparative effectiveness and retrospective study, performed in the same scenario in Cuba in the real-world population (11.9 months and 54.0%, respectively) [28].

Subramanian [29] analyzed retrospectively 15 Indian patients with unresectable, locally advanced, or metastatic ESCC, treated with nimotuzumab combined with standard treatments from October 2006 to November 2016. They achieved a 33% of complete response, and the objective response rate was 100%. Also, the 1-year survival rate (58.33%) and the median OS (26.8 months) reached for these patients were higher than the values obtained in our study.

Chen assessed clinicopathological factors related to disease-specific survival (DSS) and obtained that older age and middle esophagus location were poor prognostic factors for DSS in the United States [30]. In the Indian study, it was a lower mean age value at the time of treatment compared with the 10 years older mean age of the Cuban patient cohort. On the other hand, in the Subramanian study more than 50% (8 of 15 patients) had the tumor in the lower thoracic region, while in our study, more than 70% had the tumor located in the middle thoracic region.

Another aspect that may have contributed to obtain less survival benefit in our study with respect to the Indian cohort results is the smoking and alcoholism history of the study population. In our cohort, 65.0% were smokers, 23.0% were ex-smokers, and 79% consumed alcohol. There is sufficient evidence of the impact of these factors for the prognosis of ESCC.

Despite the fact that survival and toxicity are the most important endpoints for evaluating a treatment option in cancer, health-related quality of life (HRQoL) is also a noteworthy endpoint, which must be accounted for in larger clinical trials for this patient population. However, the studies that evaluated the role of systemic therapies in improving HRQoL in gastroesophageal cancer patients are limited.

In particular, the quality of life of patients with esophageal cancer is first negatively affected by the obstructing tumor and later by complex treatment. On the other hand, gastrointestinal toxicities deriving from targeted therapies are important issues in the oncologic setting, as they can negatively impair quality of life, reducing patient's adherence to treatment and dose intensity, so ultimately possibly affecting the outcomes. Bossi recommends to pay specific attention when analyzing and reporting the lower-grade gastrointestinal toxicities, as they may severely impact on quality of life [31].

Regarding the evaluation of the quality of life, in this study we obtained a significant increment in the general state of new diagnosis patients and a mild increment (although not significant) in their physical and social functioning. The increase in dysphagia is characteristic of patients with advanced esophageal tumors. Moreover, the favorable evolution of the general state of health of these patients, and the reduction in the appearance of nausea, insomnia, constipation, eating difficulties, and the choking when swallowing, might suggest some of the long-term benefits and consequences of nimotuzumab treatment.

In this study, the diarrhea was identified as one of the most frequent nimotuzumab-related adverse events. However, the inquiries about this symptom through the application of the QoL questionnaire proved the reduction in the prevalence of this symptom at the end of the first year of treatment, although without statistical significance.

In the NICE study, Castro employed a different scale (the Functional Assessment of Cancer Therapy General (FACT-G) questionnaire). They found no significant differences among the treatment groups in terms of the total score and the score related to the domains of social and emotional functioning. The only significant difference was observed for the subscale of physical functioning (*p*=0.03), in favor of the group treated with the concurrent therapy [16].

With this post-marketing study, we corroborate that the administration of nimotuzumab concurrent with chemoradiotherapy works in real-world circumstances. Those patients medically unfit for CT or RT (unsuitable for or who refuse surgery) did not receive the same benefit of the nimotuzumab concurrent treatment modality. From the methodological point of view, this study has the limitation of being neither controlled nor randomized because the use of nimotuzumab concurrent with chemotherapy had previously been registered as the therapy of choice in this indication. Therefore, from an ethical perspective, it was not appropriate to deprive patients from this treatment. Furthermore, the stratum of patients with disease recurrence was very small and not enough for the result generalization. Other issues that we could not evaluate need more investigation, such as the biomarkers of predictive of response to nimotuzumab, the correct time point for evaluating those biomarkers, and the immune response in ESCC patients, for selecting appropriate patients and exploring more potential mechanisms to enhance anti-EGFR efficacy.

## 5. Conclusions

The monoclonal antibody nimotuzumab was safe for patients with locally advanced or metastatic esophageal squamous cell carcinoma. New diagnosis patients treated with nimotuzumab concurrent with chemotherapy and radiotherapy reached better overall survival, progression-free survival, and quality of life than the rest of the patients treated with the monoclonal.

## Figures and Tables

**Table 1:** Distribution of patients according to baseline characteristics.

Baseline characteristics		New diagnosis			Recurrent		
		Nimo + CT + RT (*n* = 44)	Nimo + CT (*n* = 21)	Nimo + RT (*n* = 14)	Nimo (*n* = 21)	Nimo + CT (*n* = 5)	Nimo (*n* = 6)
		No. (%)	No. (%)	No. (%)	No. (%)	No. (%)	No. (%)
Gender	Male	40 (89.9)	17 (85.0)	13 (92.9)	17 (81.0)	5 (100)	6 (100)
	Female	5 (11.1)	3 (15.0)	1 (7.1)	4 (19.0)	—	—

Age	Mean ± SD	63.9 ± 6.8	60.5 ± 13.2	60.9 ± 9.6	61.9 ± 7.6	56.4 ± 5.9	64.1 ± 8.2
	Median ± *R*	64 ± 10	57 ± 23	62 ± 15	61.0 ± 11	56 ± 11	67.0 ± 16
	(Mín; máx)	(48; 77)	(42; 86)	(47; 76)	(49; 76)	(49; 64)	(53; 75)

Smoking	Nonsmoker	5 (11.1)	3 (15.0)	2 (14.3)	2 (9.5)	—	—
	Ex-smoker	10 (22.2)	2 (10.0)	5 (35.7)	6 (28.6)	1 (20.0)	2 (33.4)
	Smoker	30 (66.7)	15 (75.0)	7 (50.0)	13 (61.9)	4 (80.0)	4 (66.6)

Alcoholism	Yes	37 (82.2)	15 (75.0)	11 (78.6)	16 (76.2)	3 (60.0)	3 (50.0)
	No	8 (17.8)	5 (15.0)	3 (21.4)	5 (23.8)	2 (40.0)	3 (50.0)

Tumor location	Cervical portion	3 (6.8)	3 (14.3)	1 (7.1)	1 (4.8)	—	—
	Intrathoracic upper	6 (13.6)	5 (23.8)	4 (28.6)	3 (14.3)	—	—
	Intrathoracic middle	33 (75.0)	11 (52.4)	9 (64.3)	15 (71.4)	4 (80.0)	6 (100.0)
	Not available	2 (4.5)	2 (9.5)	—	2 (9.5)	1 (20.0)	—

Clinical stage	Ia	3 (6.8)	1 (4.8)	—	—	—	—
	Ib	1 (2.3)	4 (19.0)	1 (7.1)	—	1 (20.0)	—
	IIa	3 (6.8)	2 (9.5)	1 (7.1)	1 (4.8)	—	—
	IIb	4 (9.1)	2 (9.5)	1 (7.1)	2 (9.5)	1 (20.0)	—
	IIIa	8 (18.2)	1 (4.8)	4 (28.6)	4 (19.0)	—	1 (16.7)
	IIIb	14 (31.8)	2 (9.5)	5 (35.7)	4 (19.0)	1 (20.0)	1 (16.7)
	IIIc	1 (2.3)	1 (4.8)	—	1 (4.8)	—	1 (16.7)
	IV	8 (18.2)	6 (28.6)	2 (14.3)	7 (33.3)	2 (40.0)	3 (50.0)
	Not available	2 (9.5)	2 (9.5)	—	2 (9.5)	—	—

Nimo: nimotuzumab, CT: chemotherapy, RT: radiotherapy, SD: standard deviation, *R*: range.

**Table 2:** General information of adverse events.

Categories for patients^1^and AE	Nimotuzumab treatment modalities				
	Nimo no. (%)	Nimo + CT no. (%)	Nimo + RT no. (%)	Nimo + CT + RT no. (%)	Total no. (%)
Patients with some AE	10 (47.6)	11 (52.4)	8 (57.1)	32 (72.7)	61 (61)
Patients with some nimotuzumab-related AE	1 (4.8)	2 (9.5)	0 (0)	4 (9.1)	7 (7)
Patients with some 3-4 severity degree AE	2 (9.5)	3 (14.3)	3 (21.4)	9 (20.5)	17 (17)
Patients with some nimotuzumab-related 3-4 severity degree AE	0 (0)	1 (4.8)	0 (0)	0 (0)	1 (1)
Patients with some serious AE	3 (14.3)	7 (33.3)	2 (14.3)	15 (34.1)	27 (27)
Patients with some serious nimotuzumab-related AE	0 (0)	1 (4.8)	0 (0)	1 (2.3)	2 (2)
Adverse events	27 (100)	31 (100)	17 (100)	154 (100)	229 (100)
Nimotuzumab-related AE	2 (7.4)	6 (19.4)	0 (0)	6 (3.9)	14 (6.1)
3-4 severity degree AE	2 (7.4)	5 (16.1)	3 (17.6)	13 (8.4)	23 (10)
3-4 severity degree nimotuzumab-related AE	0 (0)	1 (3.2)	0 (0)	0 (0)	1 (0.4)
Serious AE	3 (11.1)	10 (32.3)	2 (11.8)	24 (15.6)	39 (17)
Serious AE related to nimotuzumab	0 (0)	1 (3.2)	0 (0)	2 (1.3)	3 (1.3)

Nimo: nimotuzumab, CT: chemotherapy, RT: radiotherapy, AEs: adverse events. ^1^Subjects can be included in more than one category.

**Table 3:** Response evaluation in the new diagnosis stratum.

Response evaluation (*N* = 100)		Nimo + CT + RT		Nimo + CT		Nimo + RT		Nimo		Total	
		No.	%	No.	%	No.	%	No.	%	No.	%
Response	Complete response (CR)	10	22.7	2	9.5	2	14.3	2	9.5	16	16.0
	Partial response (PR)	6	13.6	2	9.5	0	0.0	0	0.0	8	8.0
	Stable disease (SD)	2	4.5	0	0.0	1	7.1	2	9.5	5	5.0
	Progressive disease (PD)	7	15.9	4	19.0	2	14.3	2	9.5	15	15.0
	Not evaluated	19	43.2	13	61.9	9	64.3	15	71.4	56	56.0

Objective response	*CR* + *PR*	**16**	**36.3**	4	19.0	2	14.3	2	9.5	**24**	**24.0**
	*SD* + *PD*	9	20.4	4	19.0	3	21.4	4	19.0	20	20.0
	Not evaluated	19	43.2	13	61.9	9	64.3	15	71.4	56	56.0

Disease control	*CR* + *PR* + *SD*	18	**40.8**	4	19.0	3	21.4	4	19.0	**29**	**29.0**
	Progressive disease	7	15.9	4	19.0	2	14.3	2	9.5	15	15.0
	Not evaluated	19	43.2	13	61.9	9	64.3	15	71.4	56	56.0

Nimo: nimotuzumab, CT: chemotherapy, RT: radiotherapy.

**Table 4:** Quality of life's evolution over time. QLQ-C30 questionnaire.

Domain	Baseline (*N* = 87)	Treatment maintenance			*p* value
		Month 3 (*N* = 27)	Month 6 (*N* = 17)	Month 12 (*N* = 15)	
	Mean (SD)	Mean (SD)	Mean (SD)	Mean (SD)	
Global health status	56.9 (19.2)	64.1 (21.3)	63.2 (24.1)	72.8 (13.5)	**0.03** ^*∗*^
Physical functioning	80.0 (24.6)	87.2 (20.8)	86.3 (21.4)	88.4 (15.0)	0.46
Role functioning	77.6 (29.8)	87.7 (26.0)	86.3 (26.5)	86.7 (21.1)	0.23
Emotional functioning	73.9 (25.7)	76.3 (24.9)	78.4 (26.4)	81.7 (16.4)	0.54
Cognitive functioning	90.2 (21.3)	95.5 (8.9)	96.1 (7.3)	93.3 (12.3)	0.37
Social functioning	76.4 (30.7)	88.5 (15.5)	84.3 (26.7)	81.1 (30.1)	0.36
Fatigue	26.2 (25.0)	19.8 (26.6)	13.7 (21.0)	16.3 (15.1)	0.93
Nausea	13.6 (20.3)	4.3 (7.4)	3.9 (7.3)	4.4 (9.9)	**0.009** ^*∗*^
Pain	26.2 (25.7)	20.4 (26.7)	19.6 (27.8)	17.8 (23.1)	0.24
Dyspnea	15.7 (24.3)	8.6 (19.8)	5.9 (13.1)	11.1 (16.3)	0.26
Insomnia	32.6 (29.2)	17.3 (19.3)	17.6 (26.7)	15.6 (17.2)	**0.04** ^*∗*^
Appetite loss	28.4 (29.4)	23.5 (29.0)	25.5 (25.1)	24.4 (23.5)	0.12
Constipation	19.9 (30.7)	6.4 (21.1)	2.0 (8.1)	8.9 (19.8)	**0.04** ^*∗*^
Diarrhea	3.4 (10.2)	1.3 (6.5)	2.0 (8.1)	0.0 (0.0)	0.10
Financial difficulties	34.1 (33.3)	24.4 (20.1)	33.3 (31.4)	24.4 (29.5)	0.10

^*∗*^Statistically significant values according to the ANOVA test for repeated measurements. SD: standard deviation.

**Table 5:** Changes over time in specific parameters related to the quality of life in esophageal cancer. QLQ-OES-18 questionnaire.

QLQ-OES-18 domain	Baseline (*N* = 87)	Treatment maintenance			*p* Value
		Month 3 (*N* = 27)	Month 6 (*N* = 17)	Month 12 (*N* = 15)	
	Mean (SD)	Mean (SD)	Mean (SD)	Mean (SD)	
Dysphagia	39.5 (22.6)	58.0 (23.5)	53.6 (28.4)	52.6 (31.6)	**0.02** ^*∗*^
Eating difficulties	42.0 (25.9)	27.2 (27.8)	28.4 (28.1)	19.4 (23.9)	**0.0001** ^*∗*^
Reflux	23.9 (26.4)	25.6 (25.0)	28.4 (21.9)	18.9 (22.6)	0.39
Esophageal pain	26.3 (25.4)	22.6 (26.9)	20.9 (27.2)	17.0 (25.5)	0.24
Trouble swallowing saliva	25.2 (27.5)	19.8 (29.6)	15.7 (20.8)	15.6 (24.8)	0.06
Choking when swallowing	46.0 (31.4)	22.2 (27.7)	37.3 (35.1)	22.2 (30.0)	**0.0006** ^*∗*^
Dry mouth	23.0 (29.8)	9.9 (18.1)	17.6 (26.7)	20.0 (21.1)	0.66
Trouble with taste	15.7 (28.7)	17.3 (31.2)	7.8 (18.7)	6.7 (13.8)	0.49
Trouble with coughing	21.5 (27.4)	16.0 (29.8)	13.7 (23.7)	4.4 (11.7)	0.48
Speech difficulties	12.6 (26.0)	4.9 (12.1)	9.8 (15.7)	6.7 (13.8)	0.21

^*∗*^Statistically significant values according to the ANOVA test for repeated measurements. SD: standard deviation.

## Data Availability

The data sets used and/or analyzed during this study are available from the corresponding author upon reasonable request.

## References

[1] Bray F., Ferlay J., Soerjomataram I., Siegel R. L., Torre L. A., Jemal A. (2018). Global cancer statistics 2018: GLOBOCAN estimates of incidence and mortality worldwide for 36 cancers in 185 Countries. *Cancer Journal for Clincians*.

[2] Salud D., Estadístico A. (2014). Dirección de registros médicos y estadísticas de salud. *Ministerio de Salud Pública de cuba*.

[3] National Comprehensive Cancer Network (NCCN) (2019). Clinical practice guideline in oncology: esophageal and esophagogastric junction cancer. https://www.nccn.org.

[4] Ammannagari N., Atasoy A. (2018). Current status of immunotherapy and immune biomarkers in gastro-esophageal cancers. *Journal of Gastrointestinal Oncology*.

[5] Toshimitsu Yamaoka T., Kusumoto S., Ando K., Ohba M., Ohmori T. (2018). Receptor tyrosine kinase-targeted cancer therapy. *International Journal of Molecular Science*.

[6] Song J., Shi W., Zhang Y., Sun M., Liang X., Zheng S. (2016). Epidermal growth factor receptor and B7-H3 expression in esophageal squamous tissues correlate to patient prognosis. *Onco Targets Therapy*.

[7] Jiang D., Li X., Wang H. (2015). The prognostic value of EGFR overexpression and amplification in esophageal squamous cell carcinoma. *BMC Cancer*.

[8] Garrido G., Tikhomirov I. A., Rabasa A. (2011). Bivalent binding by intermediate affinity of nimotuzumab. A contribution to explain antibody clinical profile. *Cancer Biology & Therapy*.

[9] Mazorra Z., Lavastida A., Concha-Benavente F. (2017). Nimotuzumab Induces NK cell activation, cytotoxicity, dendritic cell maturation and expansion of EGFR-specific T cells in head and neck cancer patients. *Frontiers in Pharmacology*.

[10] Tundidor Y., García-Hernández C. P., Pupo A., Cabrera Infante Y., Rojas G. (2014). Delineating the functional map of the interaction between nimotuzumab and the epidermal growth factor receptor. *mAbs*.

[11] Ramos-Suzarte M., Lorenzo-Luaces P., Lazo N. G., Perez M. L., Soraino J. L. (2012). Treatment of malignant, non-resectable, epithelial origin esophageal tumours with the humanized anti-epidermal growth factor antibody Nimotuzumab combined with radiation therapy and chemotherapy. *Cancer Biology & Therapy*.

[12] Ling Y., Chen J., Tao M., Chu X., Zhang X. (2012). A pilot study of Nimotuzumab combined with cisplatin and 5-FU in patients with advanced esophageal squamous cell carcinoma. *Journal of Thoracic Disease*.

[13] Liang J., Mingyan E., Gu G., Zhao L., Li X., Xiu X. (2013). Nimotuzumab combined with radiotherapy for esophageal cancer: preliminary study of a Phase II clinical trial. *Onco Targets and Therapy*.

[14] Lu M., Wang X., Shen L., Jia J., Gong J., Li J. (2016). Nimotuzumab plus paclitaxel and cisplatin as the first line treatment for advanced esophageal squamous cell cancer: A single centre prospective phase II trial. *Cancer Science*.

[15] Zhao K., Hu X., Wu X., Fu X., Fan M., Jiang G. L. (2012). A phase I dose escalation study of nimotuzumab in combination with concurrent chemoradiation for patients with locally advanced squamous cell carcinoma of esophagus. *Invest New Drugs*.

[16] Junior G. C., Segalla J. G., Azevedo S. J., Andrade C. J., Grabarz D. (2018). A randomised phase II study of chemoradiotherapy with or without nimotuzumab in locally advanced oesophageal cancer: NICE trial. *European Journal of Cancer*.

[17] Verduzco-Rodriguez L., Aguirre-Gonzalez E. H., Verduzco-Aguirre H. C. (2011). Durable complete response induced by paclitaxel-Nimotuzumab-methotrexate chemotherapy in a patient with metastatic head and neck squamous cell carcinoma. *Hematology/Oncology Stem Cell Theropy*.

[18] Ramos T. C., Figueredo J., Catala M. (2006). Treatment of high-grade glioma patients with the humanized anti-epidermal growth factor receptor (EGFR) antibody h-R3: report from a phase I/II trial. *Cancer Biology and Theropy*.

[19] Crombet T., Cabanas R., Alert J., Valdes J. (2009). Nimotuzumab and radiotherapy in children and adolescents with brain stem glioma: preliminary results from a phase II study. *EJC Supplements*.

[20] Massimino M., Bode U., Biassoni V., Fleischhack G. (2011). Nimotuzumab for pediatric diffuse intrinsic pontine gliomas. *Expert Opinion on Biological Therapy*.

[21] Bach F., Westphal M. (2011). Current status of a phase III trial of Nimotuzumab (anti-EGF-R) in newly diagnosed glioblastoma. *Journal of Clinical Oncology*.

[22] Strumberg D., Schultheis B., Scheulen M. E. (2012). Phase II study of Nimotuzumab, a humanized monoclonal anti-epidermal growth factor receptor (EGFR) antibody, in patients with locally advanced or metastatic pancreatic cancer. *Invest New Drugs*.

[23] Lacouture M. E. (2006). Mechanisms of cutaneous toxicities to EGFR inhibitors. *Nature Review Cancer*.

[24] Mascia F., Mariani V., Girolomoni G., Pastore S. (2003). Blockade of the EGF receptor induces a deranged chemokine expression in keratinocytes leading to enhanced skin inflammation. *American Journal of Pathology*.

[25] Ayyappan S., Prabhakar D., Sharma N. (2013). Epidermal growth factor receptor (EGFR)-targeted therapies in esophagogastric cancer. *Anticancer Research*.

[26] Waddell T., Chau I., Cunningham D. (2013). Epirubicin, oxaliplatin, and capecitabine with or without panitumumab for patients with previously untreated advanced oesophagogastric cancer (REAL3): a randomised, open-label phase 3 trial. *Lancet Oncology*.

[27] Lordick F., Kang Y. K., Chung H. C. (2013). Capecitabine and cisplatin with or without cetuximab for patients with previously untreated advanced gastric cancer (EXPAND): a randomised, open-label phase 3 trial. *Lancet Oncology*.

[28] Saumell Y., Sanchez L., Gonzalez S. (2017). Overall survival of patients with locally advanced or metastatic esophageal squamous cell carcinoma treated with Nimotuzumab in the real world. *Advanced Therapy*.

[29] Subramanian S., Sridharan N., Balasundaram V., Chaudhari S. (2019). Effectiveness and tolerability of nimotuzumab in unresectable, locally advanced/metastatic esophageal cancer: Indian hospital-based retrospective evidence. *South Asian Journal of Cancer*.

[30] Chen Z., Ren Y., Du X. L. (2017). Incidence and survival differences in esophageal cancer among ethnic groups in the United States. *Oncotarget*.

[31] Bossi P., Lucchesi M., Antonuzzo A. (2015). Gastrointestinal toxicities from targeted therapies: measurement, duration and impact. *Current Opinion in Supportive and Palliative Care*.

